# Necrotizing fasciitis involving the chest and abdominal wall caused by *Raoultella planticola*

**DOI:** 10.1186/1471-2334-12-59

**Published:** 2012-03-17

**Authors:** Si-Hyun Kim, Kyoung Ho Roh, Young Kyung Yoon, Dong Oh Kang, Dong Woo Lee, Min Ja Kim, Jang Wook Sohn

**Affiliations:** 1 Department of Internal Medicine, Korea University Medical College, Seoul, Korea; 2 Department of Laboratory Medicine, Korea University Medical College, Seoul, Korea; 3 Department of Surgery, Korea University Medical College, Seoul, Korea

## Abstract

**Background:**

*Raoultella planticola *was originally considered to be a member of environmental *Klebsiella*. The clinical significance of *R. planticola *is still not well known.

**Case presentation:**

We describe the first case of necrotizing fasciitis involving the chest and abdominal wall caused by *R. planticola*. The identity of the organism was confirmed using 16S rRNA sequencing. The patient was successfully treated with the appropriate antibiotics combined with operative drainage and debridement.

**Conclusions:**

*R. planticola *had been described as environmental species, but should be suspected in extensive necrotizing fasciitis after minor trauma in mild to moderate immunocompromised patients.

## Background

*Raoultella planticola *is a Gram-negative, aerobic, non-motile, encapsulated rod bacterium [1]. Because these organisms are closely related to *Klebsiella *species, they can be easily misidentified as *Klebsiella pneumoniae *or *Klebsiella oxytoca *[2,3]. *R. planticola *is found in water, soil, and fish and was also isolated from clinical specimens and the hospital environment. However, human infections caused by *R. planticola *have been extremely rare, and the clinical significance remains uncharacterized. We describe the first case of necrotizing fasciitis involving the chest and abdominal wall caused by *R. planticola*.

## Case presentation

A 66-year-old man presented to the emergency department of University Hospital in Seoul, South Korea, complaining of severe, constant pain and bruising over the right flank for the previous 2 weeks. He had fallen, striking his right flank on the edge of the metal wastebasket approximately 4 weeks prior to presentation. At that time, he had a light abrasion on the right flank, but he did not receive any special treatment. He had a history of cardiovascular disease and poorly controlled type 2 diabetes mellitus over the past 40 years.

**Figure 1:**
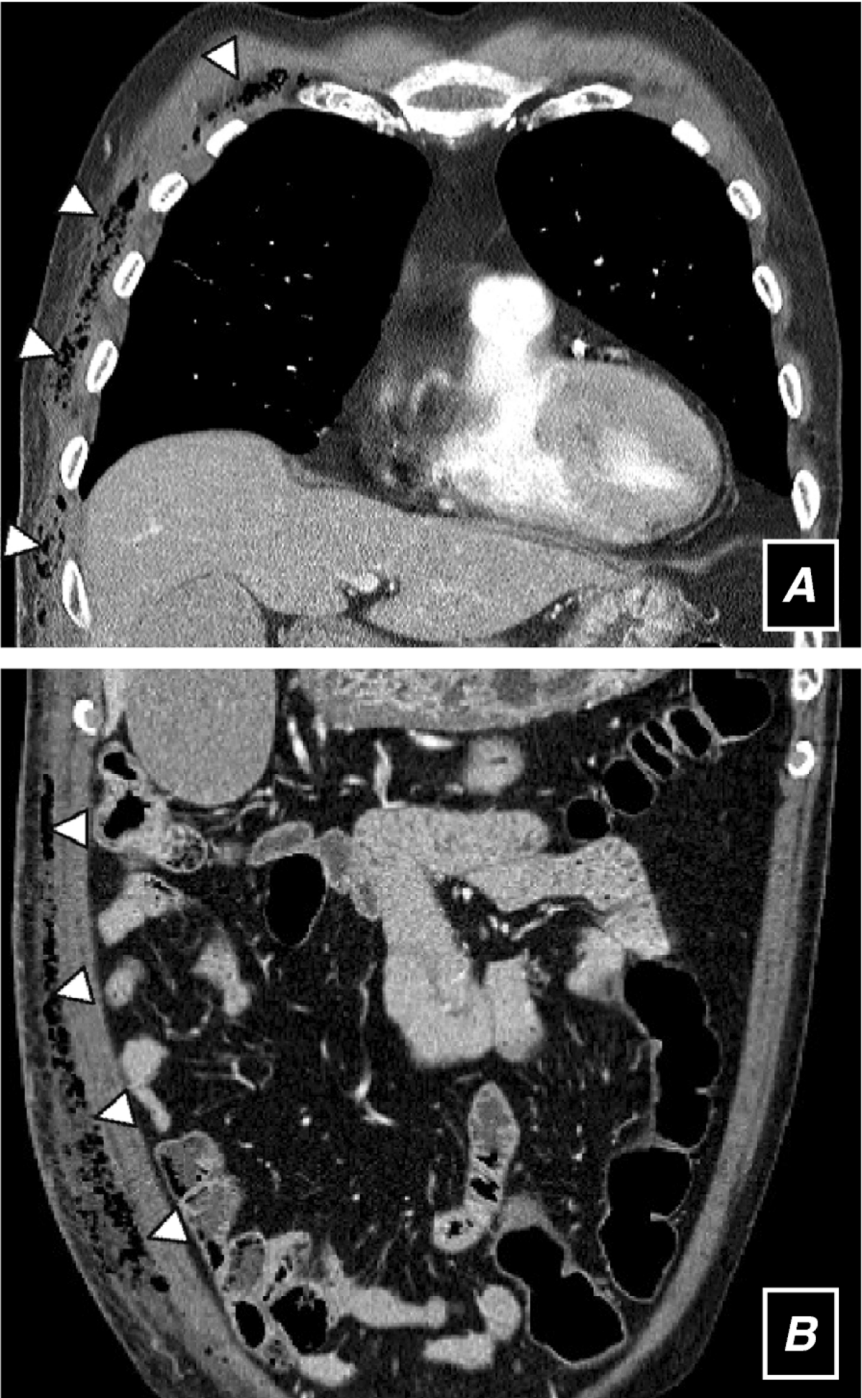
**CT scans of the chest (a) and abdomen (b) show soft tissue edema, subcutaneous fat infiltrations extending along the fascial plane, and muscular thickening with gas in the right anterolateral aspect of the chest and abdominal wall (*arrowheads*)**.

On admission, the patient's mental status was alert. His vital signs were stable except for his temperature, which was 37.7°C. Physical examination revealed intense pain on palpation, crepitus, swelling, and bruising over the right side of the abdominal wall, extending into the shoulder. No traces of the original wound remained. There were no other abnormal findings on physical examination. Laboratory tests revealed the following: white blood cell count, 8,000/mm^3 ^with 74% neutrophils (normal, 4,500-11,000/mm^3 ^with 40-75% neutrophils); hematocrit, 52.5% (normal, 38-52%); hemoglobin, 18.1 g/dL (normal, 13-17 g/dL); platelet count, 125,000/mm^3 ^(normal, 150,000-400,000/mm^3^); and C-reactive protein, 256.43 mg/L (normal, 0-3 mg/L). Computed tomography scans of the chest and abdomen revealed soft tissue edema and stranding with gas in the chest and abdominal wall (Figure 1). They did not show any abscesses in any other organs. After cultures of two blood draws and a sample obtained by direct needle aspiration were performed, treatment with cefazolin (2 g every 8 h, intravenously [i.v.]) and clindamycin (300 mg every 6 h, i.v.) was started. On hospital day 2, clindamycin was discontinued and cefazolin was changed to ceftriaxone (2 g every 24 h, i.v.) with the preliminary report of Gram-negative bacilli in the culture of the aspirated specimen. Because the patient did not show signs of systemic toxicity, elective operative drainage and debridement was performed on hospital day 3. Intraoperative findings included a foul-smelling brownish-gray exudate and subcutaneous emphysema tracking along the superficial and deep fascia from the right shoulder to the groin. On hospital day 5, the initial blood cultures were negative. The aerobic and anaerobic cultures of the needle-aspiration specimen and the necrotic tissue obtained during the operation were positive only for *R. planticola*, on hospital day 3 and 5 respectively, as determined biochemically by using the Vitek2 automated identification system (bioMérieux, Marcy l'Etoile, France; 95% probability). It demonstrated mucoid colony in both blood agar and MacConkey agar. The identity of the isolate was further confirmed using 16S rRNA sequencing [4]. Comparative sequence analysis showed a 100% identity with the sequence corresponding to the 16S rRNA gene of *R. planticola *ATCC 33531. Antibiotic susceptibility results were obtained using a Vitek2 AST-131 kit (bioMérieux) according to Clinical Laboratory Standards Institute methods. The isolate was found to be intermediate only to ampicillin and susceptible to the following antibiotics: amikacin, amoxicillin/clavulanic acid, aztreonam, cefepime, cefotaxime, cefoxitin, ceftazidime, cephalothin, gentamicin, imipenem, meropenem, piperacillin/tazobactam, tobramycin, levofloxacin, trimethoprim/sulfamethoxazole, and tigecycline. The isolates did not reveal extended-spectrum β-lactamase production.

The patient required repetitive surgical debridements, and the wound was left open because of copious amounts of tissue fluid discharge (50-150 mL daily). Despite conversion to a negative culture on hospital day 16, ceftriaxone was changed to levofloxacin (500 mg every 24 h, i.v.) because the amount of discharge did not decrease. The patient's condition was complicated by *Clostridium difficile*-associated colitis on hospital day 28. Consequently, levofloxacin was discontinued, and the patient received tigecycline (initial loading dose of 100 mg, followed by 50 mg every 12 h, i.v.) for the coverage of both *R. planticola *and *C. difficile*. Over the course of 6 weeks of antibiotic therapy combined with five surgical debridements, the patient demonstrated obvious clinical improvement of both the colitis and the necrotizing fasciitis. Wound closure was performed on hospital day 37. After the 14-day administration of tigecycline, the patient had completed the treatment and was discharged without oral antibiotics. At outpatient follow-up, 4 weeks after discharge, the surgical wound was completely healed without any evidence of necrosis. The patient reported the resolution of symptoms related to the necrotizing fasciitis of the chest and abdominal wall.

## Conclusions

Necrotizing fasciitis is usually caused by streptococci or mixed aerobe-anaerobe bacterial flora. For community-acquired infection, antimicrobial agents such as penicillin, ampicillin, and clindamycin are mainly recommended for treatment. However, very rare causes of necrotizing fasciitis include environmental species such as *Aeromonas hydrophila, Elizabethkingia meningoseptica, Chryseobacterium odoratum*, and *Serratia marcescens*. Therefore, definitive bacteriologic diagnosis based on blood or tissue specimen cultures is necessary to guide antimicrobial treatment.

*R. planticola *had been included in the genus *Klebsiella *until the late 1990's. It was originally considered to be a member of environmental *Klebsiella*, which comprises of *Klebsiella terrigena, Klebsiella ornithinolytica, Klebsiella planticola*, and *Klebsiella trevisanii*. In 1986, the last two species were combined under the name *K. planticola *because of undistinguishable phenotypic characteristics and high levels of DNA homology [5]. In 2001, *K. terrigena, K. ornithinolytica*, and *K. planticola *were transferred to the new genus *Raoultella *on the basis of 16S rRNA and *rpoB *sequences [1]. This sequence of events may previously have underestimated the role of *R. planticola *in clinical diseases.

Since *K. planticola *and *K. trevisanii *were first described in the early 1980s, only six case reports of clinical infections have been described in humans. Two cases of septicemia caused by *K. trevisanii *in the same intensive care unit after cardiac surgery were reported in 1984 and 1986, respectively [6,7]. Since then, a case of severe pancreatitis, cellulitis, surgical site infection, and two cases of bloodstream infection cause by *R. planticola *were reported separately in the past 5 years [8-11]. The first two patients with community-onset infection were not immunocompromised hosts, but required prolonged hospitalization for long-term antibiotic therapy and surgical procedures. These isolates were resistant to ampicillin or amoxicillin [8,9]. Wolcott et al. reported a case of surgical site infection diagnosed using a rapid molecular diagnostic method [10]. The two cases of bloodstream infection were developed in hospitalized patients who had received previous antimicrobial treatment, including carbapenems [11]. These isolates were non-susceptible to carbapenems, and PCR revealed the presence of *bla*_KPC_. The two patients subsequently died despite combination therapy with different antibiotic classes. In 1986, Freney et al. considered that *R. planticola *had little virulence for humans because the two cases of septicemia occurred in compromised hosts and recovered successfully after treatment. However, recent case reports, including this case, showed more serious or fatal infections by *R. planticola *with or without antimicrobial resistance.

In the present report, *R. planticola *was the primary cause of necrotizing fasciitis involving the chest and abdominal wall. The patient was successfully treated with surgical drainage and a 6-week course of proper antibiotics, including ceftriaxone, levofloxacin, and tigecycline. *R. planticola *had been described as environmental species, but may also be a human pathogen causing necrotizing fasciitis.

## Consent

Written informed consent was obtained from the patient for publication of this Case report and any accompanying images.

## Competing interests

The authors declare that they have no competing interests.

## Authors' contributions

KSH took care of the patient and drafted and revised the manuscript. YYK, SJW, KMJ, and KDO have been involved in patient clinical care and the interpretation of data. RKH performed the standard and specific microbiologic tests and the molecular genetic studies. LDO performed the surgical support in the patient clinical care. SJW reviewed the manuscript. All authors read and approved the final manuscript.

## Pre-publication history

The pre-publication history for this paper can be accessed here:

http://www.biomedcentral.com/1471-2334/12/59/prepub
